# Single-Bolus Sequential Triple-Rule-Out CT Angiography: Image Quality and Radiation Dose on Wide-Area Detector and Dual-Source CT

**DOI:** 10.3390/jcm15187166

**Published:** 2026-09-15

**Authors:** Hyun Jung Kim, Jin Woo Kim, Sung-Jin Cha, Sung Min Ko

**Affiliations:** Department of Radiology, Wonju Severance Christian Hospital, Wonju College of Medicine, Yonsei University, Wonju 26426, Republic of Korea; radkhj@yonsei.ac.kr (H.J.K.); mew@yonsei.ac.kr (J.W.K.); cktjdwls9612a@gmail.com (S.-J.C.)

**Keywords:** computed tomography angiography, acute chest pain, triple-rule-out CT angiography, pulmonary embolism, acute aortic syndrome, radiation dose

## Abstract

**Background/Objectives**: Single-pass triple-rule-out computed tomography (CT) angiography (TRO-CTA) must compromise among differing pulmonary, coronary, and aortic contrast-transit times. Wide-area detector CT (WAD-CT) and dual-source CT (DSCT) offer different coverage, temporal resolution, and dose profiles, but direct comparative evidence for a sequential single-bolus strategy is limited in selected emergency patients with overlapping concern for acute coronary syndrome, pulmonary embolism, or acute aortic syndrome. We compared territory-specific image quality and radiation dose; diagnostic accuracy was not assessed. **Methods**: This retrospective study included 114 adults (WAD-CT, *n* = 60; DSCT, *n* = 54). After test-bolus timing, one weight-based diagnostic bolus was used for sequential pulmonary, electrocardiography-synchronized coronary, and non-gated aortic acquisitions. Attenuation, noise, signal-to-noise ratio (SNR), contrast-to-noise ratio (CNR), blinded dual-reader quality scores, and radiation dose were compared by territory. **Results**: Mean overall scores across the two readers were ≥3 for every examination in all phases. WAD-CT showed higher pulmonary trunk SNR (19.5 ± 8.9 vs. 14.3 ± 3.8; *p* = 0.002), higher ascending aortic SNR (24.9 ± 11.4 vs. 14.2 ± 3.0; *p* < 0.001), lower coronary and aortic noise, and 38.1% lower total estimated dose (6.40 ± 1.72 vs. 10.34 ± 6.91 mSv; *p* < 0.001). DSCT showed higher right coronary attenuation (671.8 ± 186.7 vs. 466.4 ± 128.9 Hounsfield units; *p* < 0.001), no significant difference in right coronary SNR (*p* = 0.681), and less aortic-root pulsation artifact (*p* < 0.001). Pulmonary- and coronary-phase overall scores were comparable. **Conclusions**: Both protocols provided acceptable territory-level image quality from one diagnostic bolus. WAD-CT provided lower coronary and aortic noise and estimated radiation dose, whereas DSCT provided higher coronary attenuation and less aortic-root pulsation artifact. Diagnostic accuracy and performance in subsegmental pulmonary arteries and distal or small coronary branches remain unestablished.

## 1. Introduction

Acute chest pain is a common reason for emergency department evaluation and requires rapid differentiation of acute coronary syndrome, pulmonary embolism, and acute aortic syndrome. These disorders can present with overlapping symptoms but require fundamentally different treatment pathways [1,2]. Initial assessment uses clinical examination, electrocardiography (ECG), serial cardiac biomarkers, chest radiography, and focused bedside imaging as appropriate. When this assessment points to one principal diagnosis, a targeted examination such as coronary computed tomography angiography (CTA), CT pulmonary angiography, or aortic CTA is generally preferred. Triple-rule-out CTA (TRO-CTA) is instead reserved for selected hemodynamically stable patients in whom concern for two or more of these life-threatening causes remains unresolved [3].

Available CT strategies include separate targeted examinations and conventional single-pass TRO-CTA. Separate examinations can require repeat transport, additional contrast administration, and additional radiation if more than one vascular territory must be evaluated. A single-pass TRO-CTA acquires the thorax continuously at one compromise delay, although peak pulmonary arterial opacification occurs during right-heart transit and optimal coronary and aortic enhancements occur later during left-heart and systemic arterial transit. Biphasic or triphasic injection can broaden the enhancement window but does not eliminate differences in optimal acquisition timing and may produce dense superior vena caval or right-heart contrast that obscures adjacent cardiovascular structures [4,5,6,7].

Sequential TRO-CTA addresses this limitation by separating pulmonary, coronary, and aortic data acquisition while using the same diagnostic contrast bolus. Here, single-pass denotes one continuous thoracic acquisition at a compromise delay, whereas sequential TRO-CTA denotes three territory-specific acquisitions that follow the bolus from the pulmonary circulation to the left-sided coronary and systemic arterial circulations [4,5,6,7]. Feasibility has been demonstrated on wide-area detector CT (WAD-CT), where 16 cm coverage permits whole-heart imaging within one cardiac cycle [8,9,10,11]. Third-generation dual-source CT (DSCT) offers a different technical profile, including high temporal resolution and rapid high-pitch thoracic coverage [12,13,14]. However, dual-source systems are usually used for single-pass TRO-CTA, and direct comparative evidence for a deliberately sequential three-phase implementation is limited.

Platform-specific differences may be most relevant in the non-ECG-gated aortic phase. DSCT provides higher effective temporal resolution and faster thoracic coverage, which may reduce aortic-root pulsation artifact. WAD-CT provides whole-heart ECG-synchronized coronary coverage, including the aortic root, but uses lower-pitch non-gated pulmonary and aortic acquisitions. Territory-specific analysis is therefore more informative than a single composite score [15,16].

We therefore compared a sequential pulmonary–coronary–aortic TRO-CTA protocol performed on a 16 cm WAD-CT system and a third-generation DSCT system in emergency patients with a harmonized final-diagnosis spectrum. The platforms differ in whole-heart z-axis coverage, effective temporal resolution, and dose-modulation behavior; a direct comparison was needed to determine whether the same physiologic single-bolus sequence preserved acceptable territory-specific image quality and to characterize the resulting trade-offs. We evaluated attenuation, image noise, signal-to-noise ratio (SNR), contrast-to-noise ratio (CNR), blinded subjective image quality, and radiation dose separately for each vascular territory. The objective was to compare the image quality and dose profiles of the two deployed protocols; disease detection, diagnostic accuracy, and clinical outcomes were outside the study scope.

## 2. Materials and Methods

### 2.1. Study Design and Population

This retrospective, single-center observational study included examinations requested from the Emergency Department and identified through the Department of Radiology at Wonju Severance Christian Hospital, Wonju, Republic of Korea. The Institutional Review Board of Wonju Severance Christian Hospital approved the study (CR324049; 2 July 2024) and waived the requirement for written informed consent. The study was performed in accordance with the Declaration of Helsinki and is reported according to the Strengthening the Reporting of Observational Studies in Epidemiology (STROBE) recommendations [17].

Eligible examinations met all of the following criteria: (1) patient age of at least 18 years; (2) emergency presentation with acute chest pain, chest discomfort, or dyspnea; (3) unresolved concurrent clinical concern for at least two conditions (acute coronary syndrome, pulmonary embolism, or acute aortic syndrome) after initial clinical assessment, ECG, and cardiac biomarkers; (4) completion of the sequential pulmonary, coronary, and aortic acquisitions; and (5) availability of demographic, scanner-dose, and final-diagnosis information. The WAD-CT source cohort was examined between July 2023 and March 2024 and comprised the 71 evaluable patients reported in our previous single-platform feasibility study [8]. The DSCT source cohort comprised 54 evaluable patients examined with the corresponding sequential protocol. General exclusion criteria were known iodinated contrast hypersensitivity, estimated glomerular filtration rate below 30 mL/min/1.73 m^2^, hemodynamic instability precluding CT transfer, incomplete acquisition of any phase, or unavailable required clinical or dose information. Patients were not excluded because of heart rate, body mass index, anticipated image quality, or the measured image quality or radiation-dose outcomes.

The WAD-CT system was installed in the emergency department, and the DSCT system in the adjacent radiology department. During weekday working hours, TRO-CTA was routinely performed on WAD-CT. Outside these hours, scanner choice depended on radiologic technologist availability and concurrent workload. Neither the requesting clinician nor the radiologist assigned patients according to clinical characteristics or anticipated image quality. Allocation was therefore logistical and non-random, without deliberate clinical selection.

**Figure 1:**
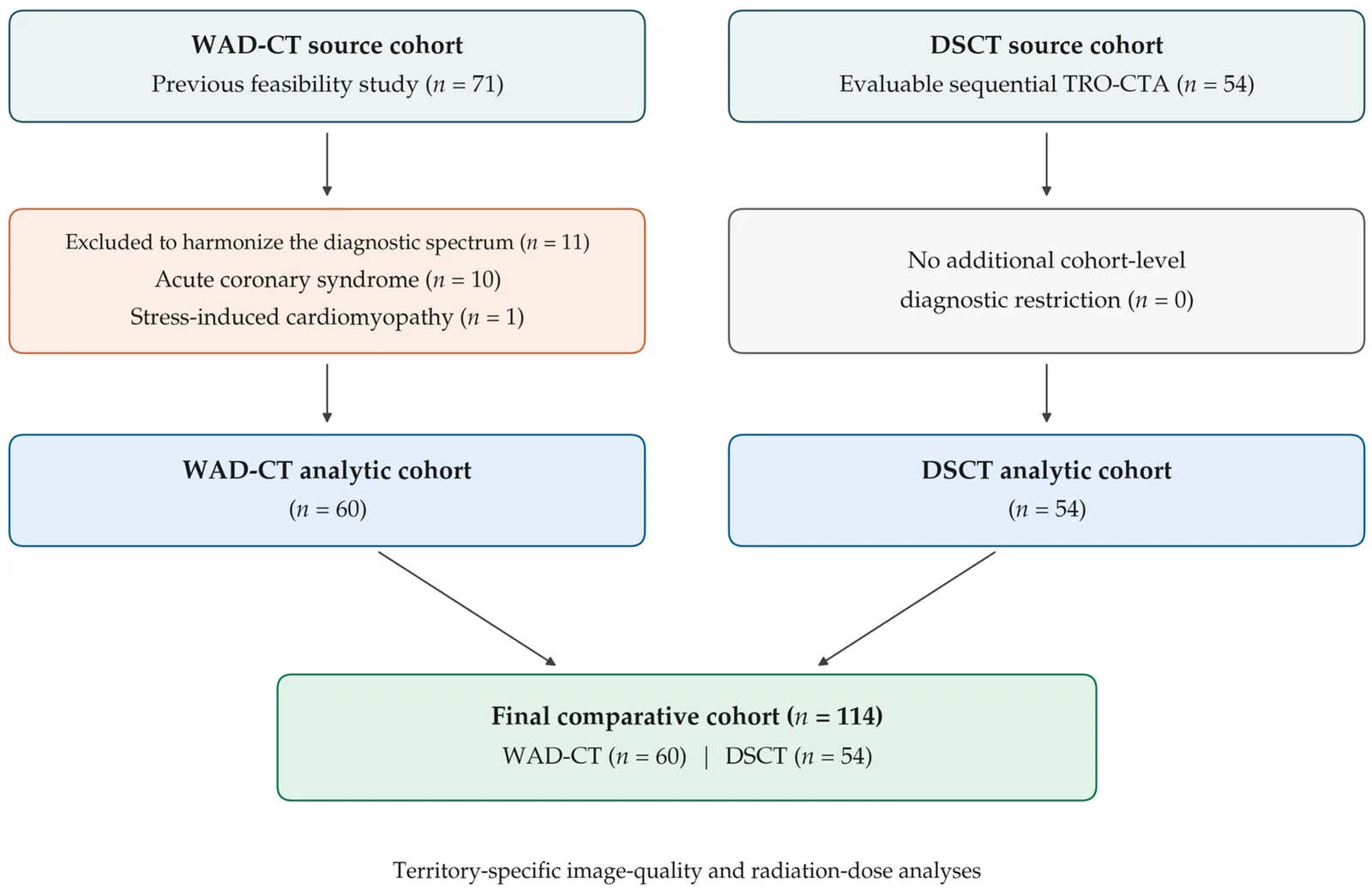
Study cohort and analytic workflow. The WAD-CT source cohort comprised 71 evaluable emergency patients from the previously published feasibility study [8]. Eleven WAD-CT patients were excluded from this comparative analysis because their final diagnostic categories were absent from the DSCT cohort: acute coronary syndrome (*n* = 10) and stress-induced cardiomyopathy (*n* = 1). The final analysis included 60 WAD-CT and 54 DSCT examinations. Image quality and radiation dose were analyzed by vascular territory. DSCT, dual-source CT; TRO-CTA, triple-rule-out CT angiography; WAD-CT, wide-area detector CT.

For the present between-platform comparison, 60 of the 71 WAD-CT patients were included. Ten patients with a final diagnosis of acute coronary syndrome and one with stress-induced cardiomyopathy were excluded because neither diagnostic category was represented in the DSCT cohort. This cohort-level diagnostic-spectrum restriction was used to improve clinical comparability between platforms; it was independent of image quality and radiation-dose results and did not involve individual patient matching or propensity-score matching. All 54 evaluable DSCT patients were included, resulting in a final analytic cohort of 114 patients (WAD-CT, *n* = 60; DSCT, *n* = 54) (Figure 1). The WAD-CT examinations therefore partially overlap with the previously reported feasibility cohort [8]. The present study addresses a distinct question by adding a complete DSCT comparison cohort, repeating territory-specific quantitative measurements, using blinded dual-reader scoring, and analyzing platform-specific radiation dose.

Final-diagnosis categories were assigned retrospectively using diagnoses and findings documented in the available clinical records. “Significant coronary artery stenosis without acute coronary syndrome” denoted significant stenosis reported on coronary CT angiography in a patient who did not have a recorded final diagnosis of acute coronary syndrome during the index episode of care. The indication for TRO-CTA was the acute presenting differential diagnosis, rather than previously established stable coronary artery disease. This category describes an eventual finding and does not imply that the stenosis caused the presenting symptoms or that a stable clinical syndrome was independently established. “No specific acute cardiothoracic finding” denoted that no specific acute cardiothoracic cause was documented after the available evaluation; it was not treated as a specific etiologic diagnosis.

### 2.2. Sequential Acquisition and Contrast Protocol

Both platforms used the same physiologic sequence: pulmonary artery phase, coronary artery phase, and thoracic aortic phase. After the separate test-bolus acquisition, the diagnostic examination used one continuous weight-based injection sequence; no additional phase-specific contrast injections were administered. The three acquisitions sampled the same diagnostic bolus during pulmonary arterial enhancement and then during systemic arterial enhancement, with the coronary acquisition preceding the dedicated thoracic aortic acquisition. Only the coronary phase was ECG-synchronized. Regions of interest for test-bolus timing were placed in the pulmonary trunk and descending thoracic aorta at the carina. The pulmonary phase started at the pulmonary trunk peak plus 2 s on WAD-CT or plus 3 s on DSCT. The coronary delay was derived from the descending aortic peak, and the aortic phase followed immediately after the coronary acquisition.

Iodinated contrast material (hexosure 350; iohexol 350 mg iodine/mL; Pharvis Pharmaceutical, Seoul, Republic of Korea) was administered through an 18–20-gauge antecubital catheter using the same protocol on both platforms. A 15 mL test injection of contrast material followed by 25 mL of saline was administered at 5.0 mL/s. The triphasic diagnostic injection comprised undiluted contrast material at 1.2 mL/kg, followed by 30 mL of a 60:40 contrast–saline mixture by volume (18 mL of contrast material and 12 mL of saline), and then a 40 mL saline chaser. All components of the diagnostic injection were delivered at 5.0 mL/s. Acquisition and reconstruction settings are detailed in Table 1.

#### 2.2.1. WAD-CT Protocol

WAD-CT examinations were performed on a 320-row system with 16 cm z-axis coverage (uCT 960+, United Imaging Healthcare, Shanghai, China). Pulmonary and aortic phases were acquired helically at 80 kVp using automatic tube-current modulation with a 66 mAs reference and pitch of 0.99. The coronary phase used prospective ECG-triggered axial acquisition at 100 kVp and 180 mAs with a 20–80% R-R acquisition window. Gantry rotation time was 0.25 s. Images were reconstructed at 0.5 mm thickness and interval with hybrid iterative reconstruction: strength 7 and C_SOFT_DA for the coronary phase, and strength 5 and B_SOFT_B for the pulmonary and aortic phases.

#### 2.2.2. DSCT Protocol

DSCT examinations were performed on a third-generation dual-source system (SOMATOM Force, Siemens Healthineers, Forchheim, Germany) operated in sequential three-phase mode rather than as a single high-pitch pass. Automated tube-voltage selection used a 100 kVp reference, and tube-current modulation used a 300 mAs reference. Pulmonary and aortic phases were acquired with pitch of 2.1. The coronary phase was ECG-synchronized with a 30–80% R-R acquisition window. Gantry rotation time was 0.25 s. Advanced modeled iterative reconstruction at strength 3 was used with 1 mm/Bv40 reconstruction for the coronary phase and 3 mm/Br44 reconstruction for pulmonary and aortic phases. Realized tube potentials were recorded for each phase.

### 2.3. Quantitative Image Analysis

A radiologist measured attenuation and standard deviation on a picture archiving and communication system workstation while blinded to the subjective scores. Circular regions of interest of 20–400 mm^2^ were placed within the vessel lumen, avoiding vessel wall, plaque, calcification, and visible artifact. Each region was measured three times and averaged. Measurements were obtained in the main pulmonary artery and ascending aorta during the pulmonary phase; aortic root, right coronary artery, left main coronary artery, and left ventricular cavity during the coronary phase; and ascending and descending thoracic aorta during the aortic phase. Same-phase epicardial or subcutaneous fat was used as the reference tissue.

Image noise was the standard deviation of attenuation within the target structure. SNR was calculated as vessel attenuation divided by vessel standard deviation. CNR was calculated as the difference between vessel and fat attenuation divided by fat standard deviation. The complete acquisition and reconstruction chain differed between systems; therefore, quantitative comparisons represent the deployed protocols rather than isolated detector performance.

### 2.4. Qualitative Image Analysis

Two radiologists with 24 and 7 years of cardiothoracic imaging experience independently reviewed examinations in random order, blinded to clinical information and each other’s scores. Scanner identifiers were removed, although complete platform blinding could not be guaranteed because reconstruction appearance differed.

Subjective image quality was scored on a five-point scale for contrast enhancement (CE), cardiac motion artifact (CM), image noise or other artifact, and overall quality. CE was assessed at the pulmonary trunk and segmental pulmonary arteries in the pulmonary phase; at the aortic root and proximal-to-mid coronary arteries in the coronary phase; and at the aortic root and distal descending aorta in the aortic phase. CM referred to motion-related blurring or duplication of cardiovascular structures. In the non-ECG-gated aortic phase, CM at the aortic root and proximal ascending aorta represented cardiac pulsation artifact. Readers were instructed to distinguish CM from insufficient CE and image noise; a higher CM score indicated less motion degradation. Aortic-phase CM was judged at the root and proximal ascending aorta, not at the descending aorta. Scores were 5, excellent; 4, good with minor non-limiting degradation; 3, adequate for interpretation; 2, poor and non-interpretable; and 1, severely degraded and non-interpretable. For between-platform summaries and acceptability rates, the two readers’ ratings were averaged for each examination and scoring domain. A mean score of at least 3 was considered technically acceptable; interobserver agreement used the individual reader ratings.

### 2.5. Radiation Dose

Volume CT dose index (CTDIvol) and dose–length product (DLP) were recorded from the scanner dose report for each phase. Effective dose was estimated as DLP multiplied by 0.014 mSv·mGy^−1^·cm^−1^ [18,19]. Total effective dose was the sum of the pulmonary, coronary, and aortic phases. These scanner-reported indices characterize protocol radiation output and provide an approximate effective-dose comparison; they do not measure patient-specific or organ-specific absorbed dose. Organ dosimetry was not available.

### 2.6. Statistical Analysis

Analyses were performed using Python 3.11 (Python Software Foundation, Wilmington, DE, USA) and IBM SPSS Statistics, version 27.0 (IBM Corp., Armonk, NY, USA). Continuous data are reported as mean ± standard deviation. Normality was assessed with the Shapiro–Wilk test; normally distributed variables were compared with Welch’s *t*-test and non-normal variables with the Mann–Whitney U test. Categorical variables are reported as numbers and percentages and were compared with the chi-square test with continuity correction or Fisher’s exact test when expected counts were below five. Because the final-diagnosis table contained several small cells, its overall between-group comparison used the Fisher–Freeman–Halton exact test. Ordinal scores are reported as median and interquartile range and compared with the Mann–Whitney U test. Standardized mean differences are reported as Cohen’s d where useful. Interobserver agreement was assessed with quadratically weighted Cohen’s kappa, interpreted as less than chance below 0, slight at 0.00–0.20, fair at 0.21–0.40, moderate at 0.41–0.60, substantial at 0.61–0.80, and almost perfect above 0.80 [20]. Because near-uniform high ratings can yield low kappa despite high absolute agreement, ceiling effects were considered when interpreting the results [21]. Sensitivity analyses used heteroscedasticity-robust linear regression adjusted for body mass index and mean heart rate. Representative outcomes were pulmonary trunk SNR, right coronary artery SNR, ascending aortic SNR, and log-transformed total effective dose. A two-sided *p*-value below 0.05 indicated statistical significance. No formal multiplicity correction was applied; isolated *p*-values near 0.05 were considered exploratory, and emphasis was placed on consistent territory-level patterns. Adjusted effect estimates for representative image quality and dose outcomes are reported with 95% confidence intervals.

### 2.7. Use of Generative Artificial Intelligence

ChatGPT (version 5.6; OpenAI, San Francisco, CA, USA) assisted with English-language editing, organization, and checks of consistency among the manuscript, tables, and reviewer responses. It was not used for patient selection or image interpretation. The authors are responsible for verifying all numerical results, references, and scientific interpretations. No identifiable patient information was included in these editing tasks. All suggested revisions were reviewed and edited by the authors, who take full responsibility for the final manuscript.

## 3. Results

### 3.1. Patient Characteristics and Realized Acquisition Parameters

The study included 60 WAD-CT and 54 DSCT examinations. Final diagnoses were acute pulmonary embolism (eight vs. five), acute aortic syndrome (four vs. two), significant coronary artery stenosis without acute coronary syndrome (seven vs. nine), pneumonia (seven vs. five), pleuritis (one vs. two), pericarditis (two vs. one), myocarditis (three vs. two), and no specific acute cardiothoracic finding (28 vs. 28) in the WAD-CT and DSCT groups, respectively (Table 2). The overall final-diagnosis distribution did not differ between groups (*p* = 0.933). Groups were also similar in age, sex, height, weight, and body mass index. Mean heart rate was higher with WAD-CT than DSCT (80.6 ± 15.4 vs. 70.2 ± 11.8 beats/min; *p* < 0.001), as was maximum heart rate (105.0 ± 40.0 vs. 78.1 ± 16.5 beats/min; *p* < 0.001). Hypertension, dyslipidemia, and current or previous smoking were more frequent in the DSCT group, whereas New York Heart Association class of at least II, chronic obstructive pulmonary disease, and diabetes did not differ.

WAD-CT tube potential was fixed at 80 kVp for pulmonary and aortic phases and 100 kVp for the coronary phase. Automated selection on DSCT produced mean realized tube potentials of 94.4 ± 19.0 kVp for the pulmonary phase, 73.1 ± 8.4 kVp for the coronary phase, and 94.8 ± 19.0 kVp for the aortic phase.

### 3.2. Attenuation and Image Noise

Pulmonary trunk attenuation did not differ between WAD-CT and DSCT (585.4 ± 254.2 vs. 514.9 ± 232.3 Hounsfield units [HU]; *p* = 0.136), and pulmonary-phase noise was also comparable (30.8 ± 6.4 vs. 35.8 ± 14.2 HU; *p* = 0.082) (Table 3). Ascending aortic attenuation during the pulmonary phase was low in both groups, consistent with right-heart timing, although its distribution differed statistically (126.5 ± 61.1 vs. 114.5 ± 84.6 HU; *p* = 0.015).

During the coronary phase, DSCT produced higher intraluminal attenuation at every measured site. Right coronary artery attenuation was 671.8 ± 186.7 HU with DSCT and 466.4 ± 128.9 HU with WAD-CT; left main attenuation was 685.5 ± 211.2 and 462.7 ± 133.6 HU, respectively; aortic root attenuation was 734.0 ± 225.5 and 474.7 ± 121.1 HU (all *p* < 0.001). The lower realized DSCT coronary tube potential was consistent with this increase in iodine attenuation.

The noise pattern was opposite. WAD-CT showed lower noise at the coronary aortic root (24.3 ± 7.3 vs. 49.7 ± 10.9 HU), right coronary artery (30.9 ± 11.6 vs. 41.9 ± 14.7 HU), left main coronary artery (30.4 ± 13.0 vs. 47.2 ± 16.5 HU), and left ventricular cavity (32.3 ± 8.7 vs. 52.6 ± 9.3 HU; all *p* < 0.001). In the aortic phase, WAD-CT provided both higher attenuation and lower noise in the ascending and descending aortas (all *p* ≤ 0.001).

### 3.3. Signal-to-Noise and Contrast-to-Noise Ratios

**Figure 2:**
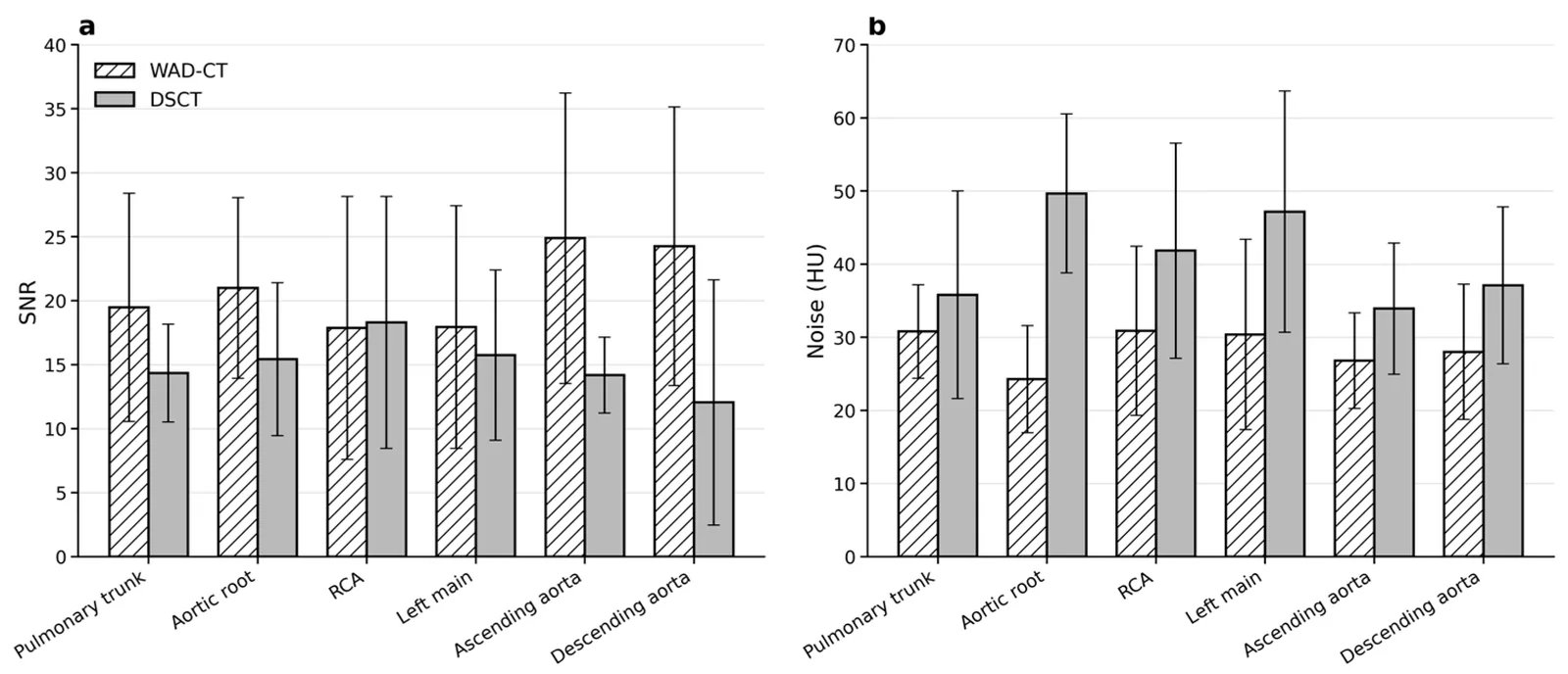
Territory-specific quantitative image quality. (**a**) Signal-to-noise ratio and (**b**) image noise at representative vascular sites, including pulmonary trunk noise in panel b. Bars show means and error bars indicate standard deviations. WAD-CT produced higher pulmonary and aortic SNR and lower noise at every coronary and aortic site; coronary SNR remained comparable because DSCT had higher attenuation. DSCT, dual-source CT; HU, Hounsfield unit; RCA, right coronary artery; SNR, signal-to-noise ratio; WAD-CT, wide-area detector CT.

WAD-CT provided higher pulmonary trunk SNR than DSCT (19.5 ± 8.9 vs. 14.3 ± 3.8; *p* = 0.002), whereas pulmonary trunk CNR did not differ (27.0 ± 14.2 vs. 22.5 ± 10.5; *p* = 0.128) (Table 3 and Figure 2). In the coronary arteries, the higher DSCT attenuation and lower WAD-CT noise largely offset each other. Right coronary artery SNR was 17.9 ± 10.3 with WAD-CT and 18.3 ± 9.8 with DSCT (*p* = 0.681); left main SNR was 17.9 ± 9.5 and 15.7 ± 6.7 (*p* = 0.330). Coronary artery CNR also did not differ significantly. Aortic root SNR was higher with WAD-CT, whereas aortic root and left ventricular CNR modestly favored DSCT. The aortic phase showed the largest quantitative WAD-CT advantage. Ascending aortic SNR was 24.9 ± 11.4 versus 14.2 ± 3.0 and descending aortic SNR was 24.3 ± 10.9 versus 12.1 ± 9.6. Corresponding CNR values were 30.8 ± 13.2 versus 18.3 ± 5.5 and 30.7 ± 13.2 versus 17.9 ± 9.2 (all *p* < 0.001).

### 3.4. Subjective Image Quality and Agreement

Every examination had a mean overall image-quality score across the two readers of at least three in every phase on both platforms (Table 4). Overall scores did not differ in the pulmonary phase (median of 4.5 [interquartile range: 4.5–5.0] vs. 5.0 [4.5–5.0]; *p* = 0.454) or coronary phase (4.5 [4.5–5.0] vs. 5.0 [4.0–5.0]; *p* = 0.913). DSCT had a higher overall aortic-phase score (5.0 [5.0–5.0] vs. 4.5 [4.4–5.0]; *p* < 0.001). These scores describe technical interpretability and do not constitute a diagnostic-accuracy endpoint.

**Figure 3:**
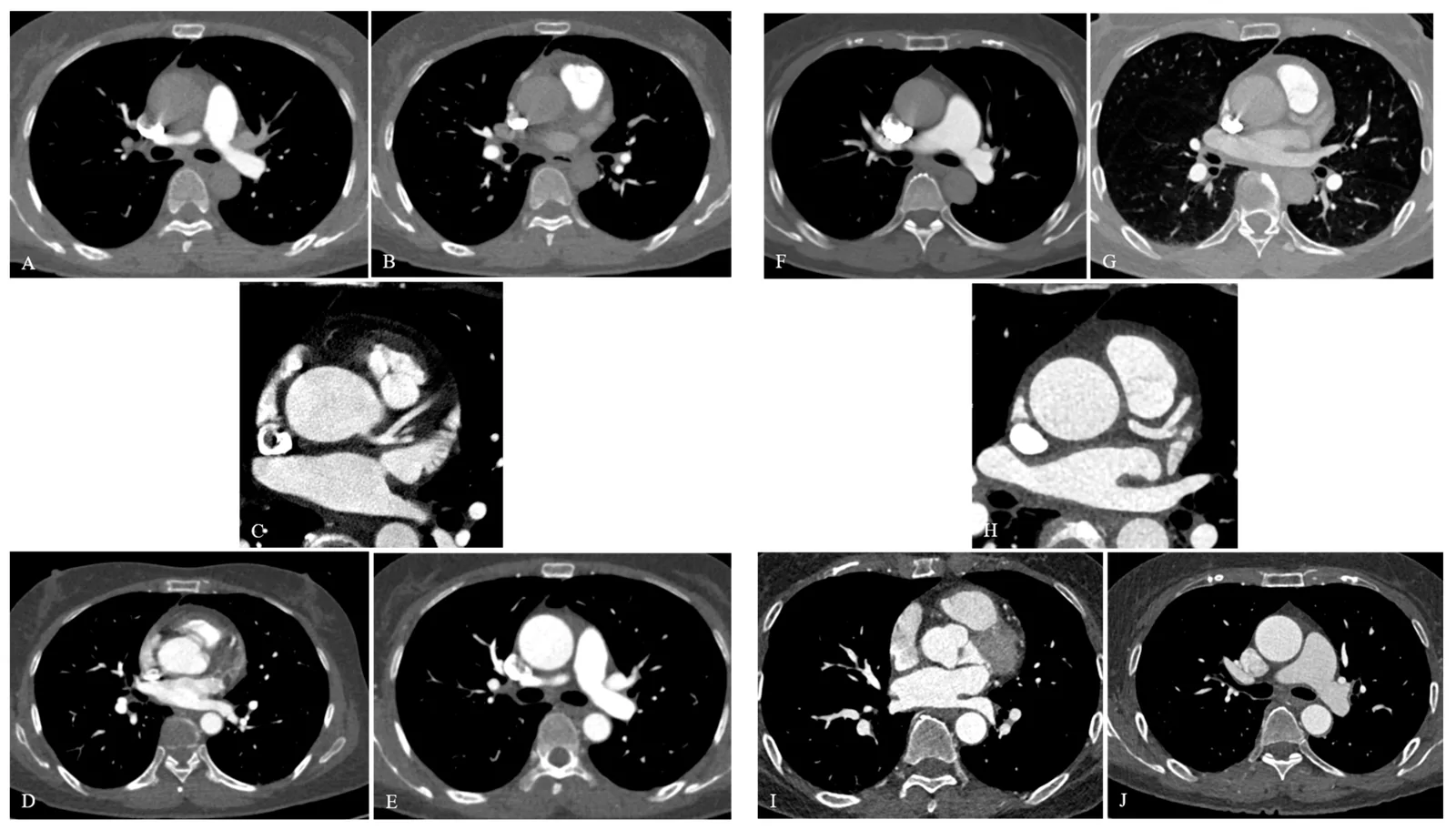
Representative sequential triple-rule-out CT angiography examinations performed with wide-area detector CT and dual-source CT. Wide-area detector CT images (**A**–**E**) show the pulmonary arterial phase (**A**,**B**), ECG-synchronized coronary phase (**C**), and non-ECG-gated thoracic aortic phase (**D**,**E**). In the aortic phase, cardiac pulsation artifact at the aortic root produces a double-contour appearance of the root and proximal ascending aorta (**D**), whereas the remaining thoracic aorta is technically assessable (**E**). Dual-source CT images (**F**–**J**) show the pulmonary arterial phase (**F**,**G**), ECG-synchronized coronary phase (H), and non-ECG-gated thoracic aortic phase (**I**,**J**). In this DSCT example, the aortic root and proximal ascending aorta are sharply delineated without appreciable cardiac motion artifact in the non-gated aortic phase (**I**), with interpretable visualization of the remaining thoracic aorta (**J**). DSCT, dual-source CT; ECG, electrocardiography.

CE did not differ between scanners in any phase. Aortic-root and distal descending aortic CE scores in the aortic phase were similar (*p* = 0.897 and *p* = 0.359, respectively). In the non-ECG-gated aortic phase, the CM score was lower with WAD-CT than DSCT (4.0 [3.5–4.5] vs. 5.0 [4.5–5.0]; *p* < 0.001; d = −1.26), reflecting greater pulsation artifact at the aortic root and proximal ascending aorta. In the ECG-synchronized coronary phase, WAD-CT had a slightly higher CM score (5.0 [4.5–5.0] vs. 4.5 [3.5–5.0]; *p* = 0.005). DSCT scored higher for pulmonary-phase image noise or other artifact (*p* < 0.001), without a difference in overall pulmonary quality. Acceptability based on the mean of the two readers’ domain scores ranged from 94.4% to 100% (Table 4). Representative images are shown in Figure 3.

Weighted kappa was substantial for coronary CE (0.70), pulmonary-phase CM (0.68), and coronary-phase CM (0.63), and moderate for aortic-phase CM (0.54) and coronary-phase aortic-root CE (0.49). Agreement was lower in domains with little score variability; kappa was near zero for pulmonary- and aortic-phase noise because almost all ratings were 4 or 5.

### 3.5. Radiation Dose and Adjusted Analyses

**Figure 4:**
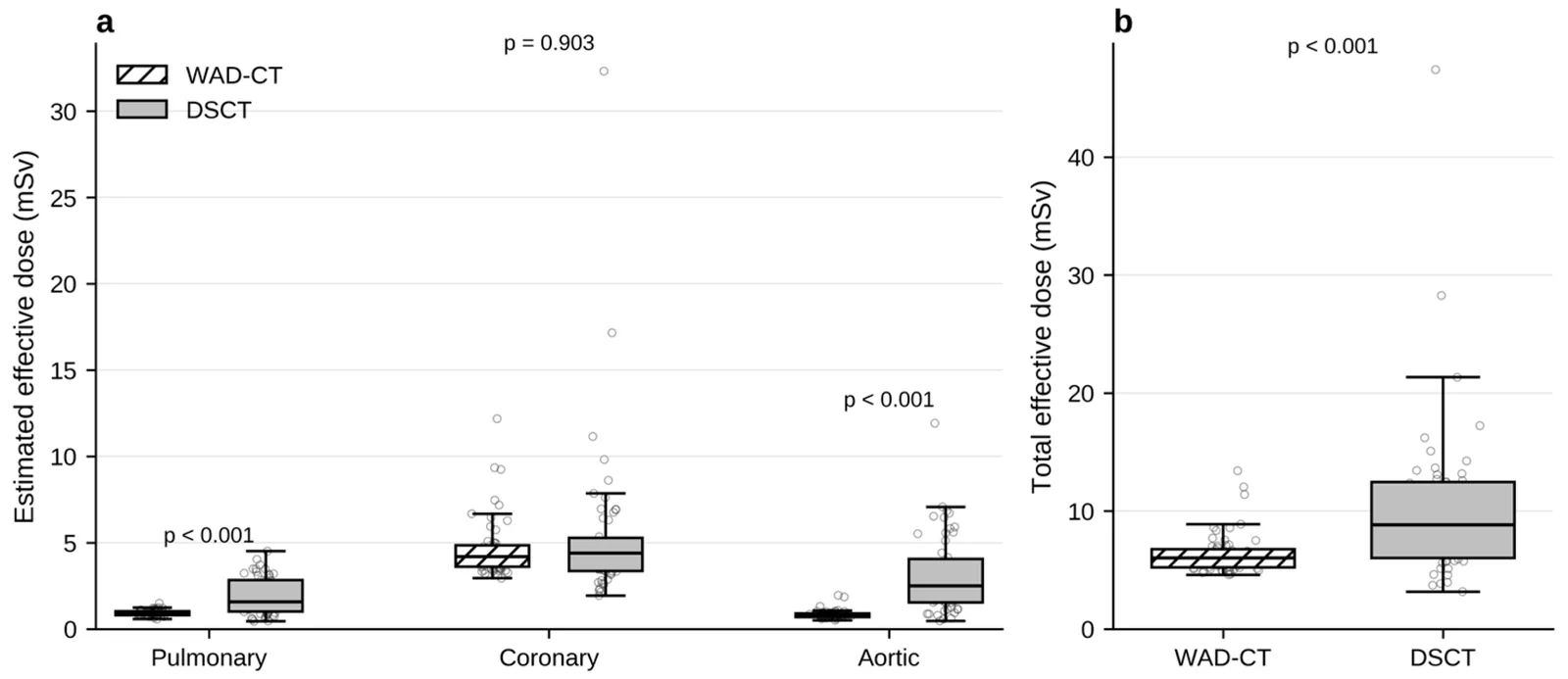
Radiation dose comparison. (**a**) Phase-specific estimated effective dose and (**b**) total examination dose. Boxes indicate the interquartile range, center lines the median, whiskers 1.5 times the interquartile range, and circles individual observations. WAD-CT had lower pulmonary, aortic, and total dose; coronary-phase dose did not differ. DSCT, dual-source CT; WAD-CT, wide-area detector CT.

Radiation data were available for all 114 examinations (Table 5 and Figure 4). WAD-CT had lower CTDIvol in the pulmonary phase (1.59 ± 0.24 vs. 3.95 ± 2.29 mGy) and aortic phase (1.52 ± 0.22 vs. 3.81 ± 2.36 mGy; both *p* < 0.001). Coronary-phase CTDIvol did not differ (20.70 ± 7.42 vs. 22.53 ± 17.64 mGy; *p* = 0.432). Estimated effective dose was lower with WAD-CT in the pulmonary phase (0.93 ± 0.16 vs. 1.89 ± 1.08 mSv) and aortic phase (0.84 ± 0.24 vs. 3.09 ± 2.24 mSv; both *p* < 0.001), but not in the coronary phase (4.63 ± 1.66 vs. 5.35 ± 4.54 mSv; *p* = 0.903). Total effective dose was 6.40 ± 1.72 mSv with WAD-CT and 10.34 ± 6.91 mSv with DSCT (*p* < 0.001), a 38.1% unadjusted reduction. The coefficients of variation were 0.27 and 0.67, respectively. After adjustment for body mass index and mean heart rate (Table 6), WAD-CT remained associated with higher pulmonary trunk SNR (adjusted difference of 5.02; 95% confidence interval [CI] 2.38–7.66; *p* < 0.001) and ascending aortic SNR (10.34; 95% CI 7.70–12.99; *p* < 0.001), while right coronary artery SNR remained similar (difference of −1.39; 95% CI −6.09 to 3.30; *p* = 0.561). WAD-CT was associated with a 26.9% lower adjusted geometric mean total effective dose (ratio of geometric means, 0.731; 95% CI 0.639–0.837; *p* < 0.001).

## 4. Discussion

Sequential pulmonary–coronary–aortic TRO-CTA provided technically acceptable mean overall image-quality scores in all three territories on both platforms. WAD-CT produced lower image noise and lower non-gated phase dose, whereas DSCT produced higher coronary attenuation and less aortic-root pulsation artifact in the non-ECG-gated aortic phase. Coronary SNR remained comparable because the attenuation and noise differences offset each other. These findings support interpretation by vascular territory and acquisition phase rather than ranking the scanners by a single composite measure.

Sequential acquisition reduces the contrast-timing compromise of conventional single-pass TRO-CTA. A dedicated pulmonary acquisition is followed by an ECG-synchronized coronary acquisition and a systemic arterial acquisition, with ECG synchronization limited to the cardiac phase. Crucially, these are not separate contrast-enhanced examinations: the three acquisitions sample the same diagnostic bolus at successive stages of cardiopulmonary transit. Previous wide-detector studies used axial or sequential strategies to reduce contrast and radiation dose while maintaining image quality [9,10,11]. The present results extend this approach to DSCT, which is more commonly used for a single high-pitch pass, and show that the same physiologic phase order can be transferred while maintaining overall image-quality acceptability.

Both platforms used the same contrast-administration protocol: a separate test bolus followed by one continuous triphasic diagnostic injection comprising undiluted contrast material, a contrast–saline mixture, and a saline chaser. No additional bolus was administered for an individual vascular territory. Individualized test-bolus timing and rapid phase transitions were required because all three acquisitions depended on the same diagnostic injection. The absence of a significant difference in overall pulmonary and coronary scores is consistent with adequate management of these timing requirements in both groups.

Pulmonary arterial enhancement was satisfactory on both platforms, and mean pulmonary trunk attenuation did not differ. This is relevant because rapid DSCT thoracic acquisitions can be limited by the amount of tube output delivered over a very short exposure, particularly at larger body size [12,14]. In the present protocol, the pulmonary arteries were examined with a dedicated, individually timed phase rather than as part of a compromise pass. The higher pulmonary trunk SNR with WAD-CT reflected a modest combination of higher attenuation and lower noise, although CNR and overall subjective quality were comparable. Thus, the quantitative difference did not produce a detectable difference in overall subjective image-quality acceptability.

The coronary phase illustrates why attenuation and noise should be interpreted together. Automated voltage selection on DSCT yielded a mean coronary tube potential of 73.1 kVp, producing substantially higher iodine attenuation than the fixed 100 kVp WAD-CT protocol. At the same time, WAD-CT had substantially lower coronary image noise. These opposing effects yielded similar SNR and CNR in the right and left main coronary arteries. Reporting attenuation alone would favor DSCT, while reporting noise alone would favor WAD-CT; the derived metrics and blinded overall scores indicate that relevant coronary quality was comparable.

The absence of a significant between-platform difference in coronary artery SNR and CNR was observed despite a mean heart rate approximately 10 beats/min higher in the WAD-CT group and greater between-patient variability in maximum heart rate. Whole-heart coverage within one prospectively triggered axial acquisition may have limited inter-beat misregistration on WAD-CT, while the broad 20–80% window allowed phase selection. DSCT benefited from high temporal resolution and lower tube potential. The adjusted analysis also found no statistically significant difference in right coronary artery SNR after accounting for body mass index and heart rate. The slightly higher coronary-phase CM score with WAD-CT may be related to single-cycle whole-heart acquisition. The differing coronary and non-gated aortic findings suggest that motion performance depends on acquisition mode as well as scanner architecture. These observations do not establish formal equivalence between the protocols or isolate detector performance under identical settings.

The aortic phase showed the clearest platform-specific trade-off. WAD-CT provided higher aortic attenuation, lower noise, and higher SNR and CNR, but DSCT had a higher overall score and less cardiac motion artifact. The motion-score difference was assessed at the aortic root and proximal ascending aorta and was consistent with cardiac pulsation artifact during a non-ECG-gated acquisition. Although both scanners used a 0.25 s gantry rotation, dual-source geometry provides higher effective temporal resolution, reducing motion-related blurring and edge duplication at the root. WAD-CT was therefore more susceptible to pulsation artifact despite adequate CE and superior quantitative vessel conspicuity [16,22]. This explains the apparent discordance between quantitative and subjective aortic-phase results.

The greater aortic-root pulsation artifact on the non-gated WAD-CT acquisition should be considered alongside the ECG-synchronized coronary dataset, which also includes the aortic root and can provide complementary assessment with less motion degradation. The coronary phase can therefore be reviewed for the root together with the dedicated aortic phase for the arch and descending aorta. Mean overall aortic image-quality scores remained technically acceptable in every examination. However, this complementary review does not by itself establish diagnostic accuracy for acute aortic lesions.

The radiation results favor the low kVp WAD-CT implementation. Total effective dose was 38% lower, and the difference arose from pulmonary and aortic phases; coronary-phase exposure was statistically similar. The fixed 80 kVp non-gated acquisitions on WAD-CT used less dose than DSCT, on which automated selection averaged approximately 94 kVp. The coronary phase accounted for 72% of the WAD-CT total dose and 52% of the DSCT total dose. The WAD-CT mean of 6.40 mSv also compares favorably with historical TRO-CTA doses that often approached or exceeded 9 mSv [4,23,24]. The smaller coefficient of variation suggests more predictable exposure. Further dose reduction should focus on the ECG-synchronized coronary phase, which contributed most of the WAD-CT total dose, while DSCT optimization should also address its relatively high aortic-phase dose.

The immediate clinical application of these findings is protocol selection and image interpretation rather than disease classification. In a hemodynamically stable patient with an overlapping differential diagnosis, either platform can implement the sequential single-bolus strategy with acceptable image quality in the vascular regions evaluated. The present WAD-CT implementation may be favored when radiation reduction is a priority, provided that the aortic root is reviewed on the ECG-synchronized coronary dataset; DSCT may be favored when minimizing aortic-root pulsation artifact is especially important. When the clinical work-up points to one predominant diagnosis, targeted coronary CT angiography, CT pulmonary angiography, or aortic CTA remains more appropriate. These practical implications do not establish sensitivity, specificity, or comparative diagnostic effectiveness.

Sequential TRO-CTA should therefore not be used indiscriminately. Patient selection requires assessment of clinical urgency, contrast-related risk, radiation exposure, and the ability to undergo CT safely. Known iodinated contrast hypersensitivity, estimated glomerular filtration rate below 30 mL/min/1.73 m^2^, and hemodynamic instability precluding CT transfer were exclusions in this study. These study exclusions should be distinguished from universal absolute contraindications in an emergency [25]. Pregnancy requires an individualized benefit–risk assessment, and inability to lie supine or cooperate with breath-holding may limit feasibility. Marked tachyarrhythmia or substantial respiratory motion may also degrade the ECG-synchronized coronary phase, although the acceptable heart-rate range depends on scanner capability and acquisition settings.

Several limitations should be considered. First, this was a retrospective single-center study with non-random scanner allocation. WAD-CT was routinely used during weekday working hours, whereas either scanner was used outside these hours according to radiologic technologist availability and workload. No clinician deliberately selected the scanner according to patient characteristics, but residual confounding remains possible. The groups differed in heart rate and cardiovascular risk factors; adjustment for body mass index and mean heart rate supported the principal quantitative findings. The cohort was too small for reliable platform-by-BMI subgroup or interaction analyses, so BMI was retained as a continuous covariate; larger studies should examine whether body size modifies comparative performance. Second, 11 patients were excluded from the previously published 71-patient WAD-CT cohort because acute coronary syndrome (*n* = 10) and stress-induced cardiomyopathy (*n* = 1) were not represented in the DSCT cohort. Although this cohort-level restriction produced similar observed final-diagnosis distributions and was independent of the image quality and dose outcomes, it was not matched individually or by propensity score and may have introduced selection bias. The findings therefore apply to the restricted diagnostic spectrum studied here. Third, tube voltage, reconstruction kernel, slice thickness, and iterative reconstruction algorithm differed between platforms. These differences represent real-world protocol performance but prevent attribution to detector geometry alone. Fourth, complete reader blinding to platform was not possible, and several qualitative domains had ceiling effects that reduced kappa. Fifth, multiple territory-level comparisons were performed without formal multiplicity correction; marginal *p*-values should therefore be interpreted cautiously. Sixth, effective dose was estimated using a generic chest conversion coefficient. Organ-specific absorbed doses, including breast dose and scattered dose to the crystalline lens, were not measured; consequently, the dose analysis compares protocol output and approximate effective dose, not tissue-specific radiation risk. Seventh, subjective assessment sampled the pulmonary trunk and segmental pulmonary arteries and the aortic root and proximal-to-mid coronary arteries. Subsegmental pulmonary arteries and distal or small coronary branches were not systematically scored, so the results cannot establish performance for subtle subsegmental pulmonary emboli or distal/small-vessel coronary lesions. Finally, the study assessed image quality rather than diagnostic accuracy. A dedicated validation study with prespecified disease endpoints and an appropriate reference standard is needed. The exclusion of patients with a final diagnosis of acute coronary syndrome also limits inference for that important target population.

## 5. Conclusions

Sequential three-phase TRO-CTA provided acceptable territory-level image quality from one diagnostic contrast bolus on both platforms. WAD-CT provided lower coronary and aortic noise and a 38% lower unadjusted estimated dose; DSCT provided higher coronary attenuation and less aortic-root pulsation artifact. These findings compare deployed protocols and can guide protocol optimization and phase-specific interpretation, but they do not establish diagnostic accuracy or clinical outcomes. Because subsegmental pulmonary arteries and distal or small coronary branches were not systematically evaluated, the protocol should not be considered validated for subtle peripheral pulmonary emboli or distal/small-vessel coronary disease.

## Figures and Tables

**Table 1:** Acquisition and reconstruction parameters of the two sequential TRO-CTA protocols.

Parameter	WAD-CT	DSCT
Scanner	uCT 960+ (United Imaging Healthcare, Shanghai, China)	SOMATOM Force (Siemens Healthineers, Forchheim, Germany)
Detector configuration	320 rows; 16 cm z-coverage	Dual-source; 2 × 192 × 0.6 mm
Acquisition order	Pulmonary → coronary → aortic	Pulmonary → coronary → aortic
Test bolus	15 mL contrast material followed by 25 mL saline	15 mL contrast material followed by 25 mL saline
Diagnostic injection	Iohexol 350 mgI/mL, 1.2 mL/kg → 30 mL of 60:40 contrast–saline mixture (18 mL contrast + 12 mL saline) → 40 mL saline	Iohexol 350 mgI/mL, 1.2 mL/kg → 30 mL of 60:40 contrast–saline mixture (18 mL contrast + 12 mL saline) → 40 mL saline
Injection rate	5.0 mL/s	5.0 mL/s
Test-bolus timing	Pulmonary trunk peak +2 s; coronary delay from descending aortic peak; aortic phase immediately after coronary phase	Pulmonary trunk peak +3 s; coronary delay from descending aortic peak; aortic phase immediately after coronary phase
Pulmonary phase	Helical; 80 kVp; 66 mAs reference; pitch, 0.99; 0.5 mm HIR level 5/B_SOFT_B	Helical; automated kVp and current; pitch, 2.1; 3 mm ADMIRE level 3/Br44
Coronary phase	Prospective ECG-triggered axial; 100 kVp; 180 mAs; 20–80% R–R; 0.5 mm HIR level 7/C_SOFT_DA	ECG-synchronized; automated kVp and current; 30–80% R–R; 1 mm ADMIRE level 3/Bv40
Aortic phase	Helical; 80 kVp; 66 mAs reference; pitch, 0.99; 0.5 mm HIR level 5/B_SOFT_B	Helical; automated kVp and current; pitch, 2.1; 3 mm ADMIRE level 3/Br44
Gantry rotation time	0.25 s	0.25 s
Realized kVp (PA/CA/TA)	80/100/80	94.4 ± 19.0/73.1 ± 8.4/94.8 ± 19.0

ADMIRE, Advanced modeled iterative reconstruction; CA, coronary artery phase; CT, computed tomography; DSCT, dual-source CT; ECG, electrocardiography; HIR, hybrid iterative reconstruction; PA, pulmonary artery phase; TA, thoracic aortic phase; TRO-CTA, triple-rule-out CT angiography; WAD-CT, wide-area detector CT.

**Table 2:** Patient characteristics and final diagnoses.

	WAD-CT (*n* = 60)	DSCT (*n* = 54)	*p*-Value
Age, years	68.7 ± 17.3	72.3 ± 12.2	0.566
Female sex	28 (46.7%)	23 (42.6%)	0.804
Height, cm	160.8 ± 9.5	159.7 ± 11.2	0.595
Weight, kg	62.7 ± 13.7	63.2 ± 13.5	0.839
Body mass index, kg/m^2^	24.06 ± 3.74	24.64 ± 3.84	0.417
Minimum heart rate, beats/min	68.6 ± 15.9	64.7 ± 13.1	0.283
Maximum heart rate, beats/min	105.0 ± 40.0	78.1 ± 16.5	<0.001
Mean heart rate, beats/min	80.6 ± 15.4	70.2 ± 11.8	<0.001
NYHA functional class ≥ II	17 (28.3%)	8 (14.8%)	0.130
Chronic obstructive pulmonary disease	3 (5.0%)	4 (7.4%)	0.706
Hypertension	35 (58.3%)	42 (77.8%)	0.044
Diabetes mellitus	16 (26.7%)	16 (29.6%)	0.886
Dyslipidemia	20 (33.3%)	39 (72.2%)	<0.001
Current or previous smoking	11 (18.3%)	22 (40.7%)	0.015
Final diagnosis			0.933
Acute pulmonary embolism	8 (13.3%)	5 (9.3%)	
Acute aortic syndrome	4 (6.7%)	2 (3.7%)	
Significant coronary artery stenosis without acute coronary syndrome	7 (11.7%)	9 (16.7%)	
Pneumonia	7 (11.7%)	5 (9.3%)	
Pleuritis	1 (1.7%)	2 (3.7%)	
Pericarditis	2 (3.3%)	1 (1.9%)	
Myocarditis	3 (5.0%)	2 (3.7%)	
No specific acute cardiothoracic finding	28 (46.7%)	28 (51.9%)	

Values are means ± standard deviations or numbers (percentages). The *p*-value for the overall final-diagnosis distribution was calculated using the Fisher–Freeman–Halton exact test. Significant coronary artery stenosis without acute coronary syndrome indicates significant stenosis reported on coronary CT angiography without a recorded final diagnosis of acute coronary syndrome during the index episode of care. No specific acute cardiothoracic finding indicates that no specific acute cardiothoracic cause was documented after the available evaluation; it is not a specific etiologic diagnosis. DSCT, dual-source CT; NYHA, New York Heart Association; WAD-CT, wide-area detector CT.

**Table 3:** Territory-specific attenuation, image noise, signal-to-noise ratio, and contrast-to-noise ratio.

Phase	Target	Measure	WAD-CT (*n* = 60)	DSCT (*n* = 54)	*p*-Value	Cohen’s d
Pulmonary	PT	Attenuation, HU	585.4 ± 254.2	514.9 ± 232.3	0.136	0.29
Pulmonary	PT	Noise, HU	30.8 ± 6.4	35.8 ± 14.2	0.082	−0.47
Pulmonary	PT	SNR	19.48 ± 8.91	14.34 ± 3.83	0.002	0.74
Pulmonary	PT	CNR	27.03 ± 14.24	22.52 ± 10.54	0.128	0.36
Pulmonary	AA	Attenuation, HU	126.5 ± 61.1	114.5 ± 84.6	0.015	0.16
Coronary	AR	Attenuation, HU	474.7 ± 121.1	734.0 ± 225.5	<0.001	−1.45
Coronary	AR	Noise, HU	24.26 ± 7.33	49.67 ± 10.88	<0.001	−2.77
Coronary	AR	SNR	21.00 ± 7.06	15.43 ± 5.97	<0.001	0.85
Coronary	AR	CNR	21.22 ± 7.87	25.54 ± 10.78	0.027	−0.46
Coronary	RCA	Attenuation, HU	466.4 ± 128.9	671.8 ± 186.7	<0.001	−1.29
Coronary	RCA	Noise, HU	30.87 ± 11.56	41.85 ± 14.71	<0.001	−0.84
Coronary	RCA	SNR	17.87 ± 10.27	18.30 ± 9.84	0.681	−0.04
Coronary	RCA	CNR	20.88 ± 7.84	23.24 ± 8.14	0.099	−0.30
Coronary	LM	Attenuation, HU	462.7 ± 133.6	685.5 ± 211.2	<0.001	−1.28
Coronary	LM	Noise, HU	30.38 ± 13.02	47.17 ± 16.50	<0.001	−1.14
Coronary	LM	SNR	17.94 ± 9.48	15.74 ± 6.65	0.330	0.27
Coronary	LM	CNR	20.83 ± 8.41	23.88 ± 9.88	0.081	−0.33
Coronary	LV cavity	Attenuation, HU	458.4 ± 125.7	718.4 ± 185.7	<0.001	−1.66
Coronary	LV cavity	Noise, HU	32.27 ± 8.65	52.64 ± 9.26	<0.001	−2.28
Coronary	LV cavity	SNR	15.22 ± 5.74	13.94 ± 4.09	0.295	0.25
Coronary	LV cavity	CNR	20.65 ± 8.20	24.80 ± 8.67	0.005	−0.49
Aortic	AA	Attenuation, HU	631.2 ± 206.7	480.5 ± 171.4	<0.001	0.79
Aortic	AA	Noise, HU	26.80 ± 6.52	33.92 ± 8.98	<0.001	−0.91
Aortic	AA	SNR	24.88 ± 11.35	14.18 ± 2.97	<0.001	1.26
Aortic	AA	CNR	30.84 ± 13.19	18.30 ± 5.51	<0.001	1.22
Aortic	DA	Attenuation, HU	625.4 ± 204.8	471.8 ± 272.1	<0.001	0.64
Aortic	DA	Noise, HU	28.01 ± 9.23	37.11 ± 10.73	<0.001	−0.91
Aortic	DA	SNR	24.26 ± 10.88	12.05 ± 9.59	<0.001	1.19
Aortic	DA	CNR	30.67 ± 13.24	17.92 ± 9.22	<0.001	1.11

Values are means ± standard deviations. Cohen’s d was calculated as WAD-CT minus DSCT divided by the pooled standard deviation. Positive values indicate higher values with WAD-CT; negative values indicate higher values with DSCT. For noise, lower values indicate better image quality. CNR, contrast-to-noise ratio; DSCT, dual-source CT; HU, Hounsfield unit; LM, left main coronary artery; LV, left ventricular cavity; RCA, right coronary artery; PT, pulmonary trunk; AA, ascending aorta; DA, descending aorta; AR, aortic root; SNR, signal-to-noise ratio; WAD-CT, wide-area detector CT.

**Table 4:** Subjective image-quality scores and interobserver agreement.

Phase	Domain	WAD-CT	DSCT	*p*-Value	Acceptable, %	Weighted κ
					WAD/DSCT	
PA	CE: pulmonary trunk	5.0 (4.5–5.0)	5.0 (4.5–5.0)	0.269	100.0/100.0	0.33
PA	CE: segmental pulmonary arteries	4.5 (4.0–5.0)	4.5 (4.0–5.0)	0.347	98.3/94.4	0.46
PA	CM	5.0 (5.0–5.0)	5.0 (5.0–5.0)	0.244	100.0/98.1	0.68
PA	Image noise/other artifact	4.5 (4.0–4.5)	4.5 (4.5–5.0)	<0.001	100.0/100.0	−0.03
CA	CE: aortic root	5.0 (4.5–5.0)	5.0 (4.5–5.0)	0.390	100.0/100.0	0.49
CA	CE: coronary arteries	5.0 (4.5–5.0)	5.0 (4.1–5.0)	0.354	100.0/94.4	0.70
CA	CM	5.0 (4.5–5.0)	4.5 (3.5–5.0)	0.005	100.0/100.0	0.63
CA	Image noise/other artifact	5.0 (4.5–5.0)	5.0 (4.5–5.0)	0.493	100.0/100.0	0.38
TA	CE: aortic root	5.0 (5.0–5.0)	5.0 (5.0–5.0)	0.897	100.0/100.0	0.35
TA	CE: distal descending aorta	5.0 (4.5–5.0)	5.0 (4.5–5.0)	0.359	100.0/100.0	0.18
TA	CM	4.0 (3.5–4.5)	5.0 (4.5–5.0)	<0.001	98.3/100.0	0.54
TA	Image noise/other artifact	4.5 (4.5–5.0)	5.0 (4.5–5.0)	0.051	100.0/100.0	−0.03
PA	Overall	4.5 (4.5–5.0)	5.0 (4.5–5.0)	0.454	100.0/100.0	0.27
CA	Overall	4.5 (4.5–5.0)	5.0 (4.0–5.0)	0.913	100.0/100.0	0.49
TA	Overall	4.5 (4.4–5.0)	5.0 (5.0–5.0)	<0.001	100.0/100.0	0.29

Scores are medians with interquartile ranges and represent the mean of the two readers’ ratings. Technical acceptability was defined as a mean score ≥ 3 across the two readers for each examination and domain; this does not require both individual ratings to be ≥3. ECG, electrocardiography; CE, contrast enhancement; CM, cardiac motion artifact. In the non-ECG-gated thoracic aortic phase, aortic-root CM represents cardiac pulsation artifact. Quadratically weighted Cohen’s kappa, pooled across the two scanner groups, is reported for interobserver agreement. CA, coronary artery phase; DSCT, dual-source CT; PA, pulmonary artery phase; TA, thoracic aortic phase; WAD-CT, wide-area detector CT.

**Table 5:** Radiation dose by acquisition phase.

Phase	Dose Metric	WAD-CT (*n* = 60)	DSCT (*n* = 54)	*p*-Value	Reduction with WAD-CT
Pulmonary phase	CTDIvol, mGy	1.59 ± 0.24	3.95 ± 2.29	<0.001	—
Coronary phase	CTDIvol, mGy	20.70 ± 7.42	22.53 ± 17.64	0.432	—
Aortic phase	CTDIvol, mGy	1.52 ± 0.22	3.81 ± 2.36	<0.001	—
Pulmonary phase	DLP, mGy·cm	66.7 ± 11.7	135.3 ± 77.1	<0.001	—
Coronary phase	DLP, mGy·cm	330.5 ± 118.8	382.4 ± 324.0	0.903	—
Aortic phase	DLP, mGy·cm	59.9 ± 17.5	220.5 ± 160.0	<0.001	—
Pulmonary phase	Effective dose, mSv	0.93 ± 0.16	1.89 ± 1.08	<0.001	50.70%
Coronary phase	Effective dose, mSv	4.63 ± 1.66	5.35 ± 4.54	0.903	—
Aortic phase	Effective dose, mSv	0.84 ± 0.24	3.09 ± 2.24	<0.001	72.80%
Total examination	Effective dose, mSv	6.40 ± 1.72	10.34 ± 6.91	<0.001	38.10%

Values are means ± standard deviations. Effective dose was estimated as dose–length product × 0.014 mSv·mGy^−1^·cm^−1^. CTDIvol, volume CT dose index; DSCT, dual-source CT; WAD-CT, wide-area detector CT.

**Table 6:** Adjusted between-platform effects for representative image quality and dose outcomes.

Phase	Representative Outcome	Adjusted Effect (WAD-CT vs. DSCT)	95% CI	*p*-Value
Pulmonary	Pulmonary trunk SNR	Mean difference, 5.02	2.38 to 7.66	<0.001
Coronary	Right coronary artery SNR	Mean difference, −1.39	−6.09 to 3.30	0.561
Aortic	Ascending aortic SNR	Mean difference, 10.34	7.70 to 12.99	<0.001
Total examination	Estimated effective dose	Ratio of geometric means, 0.731	0.639 to 0.837	<0.001

Models used heteroscedasticity-robust standard errors and were adjusted for body mass index and mean heart rate. Positive SNR mean differences favor WAD-CT. Total effective dose was log-transformed; the effect is shown as the ratio of geometric means, for which a value below 1 favors WAD-CT. CI, confidence interval; DSCT, dual-source CT; SNR, signal-to-noise ratio; WAD-CT, wide-area detector CT.

## Data Availability

The anonymized data supporting the findings of this study are available from the corresponding author upon reasonable request, subject to institutional and ethical restrictions.

## Abbreviations

The following abbreviations are used in this manuscript:

AA	Ascending aorta
ADMIRE	Advanced modeled iterative reconstruction
AR	Aortic root
CA	Coronary artery phase
CE	Contrast enhancement
CI	Confidence interval
CM	Cardiac motion artifact
CNR	Contrast-to-noise ratio
CT	Computed tomography
CTDIvol	Volume CT dose index
DA	Descending aorta
DLP	Dose–length product
DSCT	Dual-source CT
ECG	Electrocardiography
HIR	Hybrid iterative reconstruction
HU	Hounsfield unit
LM	Left main coronary artery
LV	Left ventricular cavity
NYHA	New York Heart Association
PA	Pulmonary artery phase
PT	Pulmonary trunk
RCA	Right coronary artery
SNR	Signal-to-noise ratio
STROBE	Strengthening the Reporting of Observational Studies in Epidemiology
TA	Thoracic aortic phase
TRO-CTA	Triple-rule-out computed tomography angiography
WAD-CT	Wide-area detector CT
